# Meridional anisotropy in contrast sensitivity and visual evoked potential in adults with high myopic astigmatism

**DOI:** 10.3389/fnins.2024.1457297

**Published:** 2025-01-09

**Authors:** Siu Sang Anthony Wu, Tsz Wing Leung

**Affiliations:** ^1^School of Optometry, The Hong Kong Polytechnic University, Hung Hom, Hong Kong SAR, China; ^2^Centre for Eye and Vision Research Limited, Shatin, Hong Kong SAR, China; ^3^Research Centre for Sharp Vision, The Hong Kong Polytechnic University, Hung Hom, Hong Kong SAR, China

**Keywords:** astigmatism, meridional anisotropy, meridional visual deficits, contrast sensitivity, visual evoked potential

## Abstract

**Purpose:**

Astigmatism can lead to meridional amblyopia, an orientation-specific visual deficit. This study investigated the effects of astigmatism on meridional anisotropy in contrast sensitivity (CS) and steady-state visual evoked potential (ssVEP) across a range of spatial frequencies.

**Methods:**

Thirty-two young adults with a best-corrected distance visual acuity of logMAR 0 or better were categorized into two groups: highly astigmatic (HAS, *n* = 16) with spherical-equivalent error (SE) ≥ −6.00 D and a cylindrical error (Cyl) ≥ 2.00 DC, and non-astigmatic (NAS, *n* = 16) with SE ≥ −6.00 D but Cyl ≤ 0.50 DC. We assessed CS using a spatial four-alternative forced-choice procedure and recorded ssVEP at spatial frequencies ranging from 0.6 to 12 cycles per degree (cpd) for horizontal and vertical gratings. The Area Under Log Contrast Sensitivity Function (AULCSF) and spatial frequency cutoff for the CS were also calculated.

**Results:**

The HAS group exhibited significantly lower CS for horizontal compared to vertical gratings at most spatial frequencies (*p* < 0.045 for 0.6–6 cpd), also reflected in a lower AULCSF (*p* = 0.01). This meridional anisotropy in CS was absent in the NAS group for both AULCSF and individual spatial frequencies, except at 0.6 cpd (*p* = 0.005). Spatial frequency cutoff did not differ between orientations for either group (*p* > 0.94). Conversely, ssVEP amplitudes were consistently lower for horizontal than vertical gratings in both groups, regardless of the presence of astigmatism (*p* < 0.05).

**Conclusion:**

Meridional anisotropy in contrast sensitivity was observed only in highly astigmatic participants, whereas meridional anisotropy in ssVEP was present in both highly astigmatic and non-astigmatic groups. This discrepancy between psychophysical and electrophysiological measures may be related to the static versus flickering nature of the stimuli and warrants further investigation.

## 1 Introduction

Refractive astigmatism is a prevalent refractive error, affecting over 20% (cylindrical errors >0.75 DC) of Native American and Chinese children (Zhang et al., 2023; Hashemi et al., 2018; Yoon et al., 2011; Wong et al., 2022). If not corrected during childhood, astigmatism can significantly disrupt normal vision development and increase the risk of visual impairment. For instance, children with 1.00 DC of astigmatism exhibit double the risk of visual impairment, defined as best-corrected distance visual acuity worse than 0.2 logMAR, compared to non-astigmatic children (Wang et al., 2018). This risk escalates more than eightfold with astigmatism of 3.00 DC or higher (Wang et al., 2018). Due to its orientation-dependent optical properties (Read et al., 2014), astigmatism often leads to meridional anisotropy, characterized by a significant reduction in spatial vision along the meridian most blurred by astigmatism. This meridional anisotropy is commonly observed in psychophysical assessments of grating acuity and contrast sensitivity, especially at spatial frequencies of 6 cycles per degree (cpd) or higher (Harvey et al., 2007; Mitchell and Wilkinson, 1974; Freeman and Thibos, 1975a; Leung et al., 2021). While such assessments show that the meridional anisotropy observed in individuals with high astigmatism often aligns with their astigmatic axis (Harvey et al., 2007; Mitchell and Wilkinson, 1974; Freeman and Thibos, 1975a), electrophysiological investigations using Visual Evoked Potentials (VEPs) in the primary visual cortex have yielded inconsistent results.

Earlier research using VEPs identified meridional anisotropy in astigmatic children, reporting reduced VEP amplitude along the meridian experiencing the most significant blur (Fiorentini and Maffei, 1973). While subsequent research has observed a reduction in VEP amplitude for horizontal gratings, this finding is not unique to children with refractive astigmatism but also occurs in non-astigmatic children (Yap et al., 2019; Yap et al., 2020). Indeed, recent VEP studies found no significant differences in meridional anisotropy when comparing astigmatic individuals to non-astigmatic controls (Yap et al., 2019), or when comparing astigmatic individuals with and without amblyopia (Yap et al., 2021; Gu et al., 2021). This reduced VEP amplitude for horizontal gratings (Yap et al., 2019, Yap et al., 2020) may reflect the “horizontal effect,” characterized by poorer processing of horizontal stimuli compared to oblique or vertical ones (Yap et al., 2024; Yap et al., 2019). This raises the question of whether the observed meridional anisotropy in astigmatism is a consequence of the astigmatic blur or a manifestation of this more general horizontal effect. Methodological variations, particularly the spatial frequencies employed and the specific refractive characteristics of participants, may also contribute to the observed discrepancies between psychophysical and electrophysiological findings.

Previous VEP studies investigating meridional anisotropy usually assessed cortical responses at spatial frequencies not exceeding 4 cpd (Fiorentini and Maffei, 1973; Yap et al., 2019; Yap et al., 2020). One possible explanation for this choice is the proximity of 4 cpd to the peak of normal contrast sensitivity function (Yap and Boon, 2020). Moreover, astigmatism-induced meridional anisotropies also tend to be more pronounced at this spatial frequency (Freeman and Thibos, 1975b). However, optical blur induced by astigmatism disproportionately affects high-frequency spatial vision (De Valois and De Valois, 1980). As demonstrated by psychophysical studies, meridional anisotropy in astigmatic eyes is also observed at higher spatial frequencies (≥ 6 cpd) (Harvey et al., 2007; Mitchell and Wilkinson, 1974; Freeman, 1975b; Freeman and Thibos, 1975a). This raises a critical question: could incorporating higher spatial frequencies in VEP assessments reveal more significant meridional anisotropy, thereby aligning electrophysiological data more closely with psychophysical observations? This question holds significant clinical relevance, as VEPs, which directly measure cortical electrophysiological activity, are valuable tools for evaluating vision, particularly in non-verbal individuals such as infants (Taylor and Mcculloch, 1992; Shepherd et al., 1999).

Furthermore, previous VEP studies may have exhibited variability in their findings due to the potential influence of coexisting spherical refractive errors on astigmatic blur patterns and their subsequent impact on meridional anisotropy. For example, uncorrected with-the-rule (WTR) astigmatism typically induces greater optical blur along the horizontal meridian when combined with myopia, potentially leading to a more pronounced visual deficit for horizontally oriented stimuli (Mitchell et al., 1973). Conversely, when WTR astigmatism coexists with hyperopia, the vertical meridian is predominantly affected (Mitchell et al., 1973; Harvey, 2009). This variability underscores how identical astigmatic orientations can result in distinct blur patterns depending on the accompanying spherical refractive error. The pattern of meridional anisotropy in myopic astigmatism tends to be consistent (Harvey, 2009; Mitchell et al., 1973), likely due to relatively stable blur pattern, particularly for distance vision. While working distance and accommodation can influence this anisotropy (Yap et al., 2019), the effects are less pronounced than in hyperopic astigmatism. In hyperopic astigmatism, accommodation affects the blur pattern for both distance and near vision, causing shifts in the astigmatic foci along the visual axis, which probably explains the more varied meridional anisotropy in this refractive group (Harvey et al., 2003; Freeman, 1975a). The relatively more stable and consistent pattern of optical blur in myopic astigmatism, compared to other astigmatic subtypes, leads us to focus on this refractive group for investigating meridional anisotropy in contrast sensitivity and VEP. Recent studies that have included astigmatic participants with both myopia and hyperopia (Yap et al., 2019; Yap et al., 2020; Gu et al., 2021) may introduce greater variability in their findings, potentially obscuring the effects of astigmatic blur on meridional anisotropy. Therefore, controlling for coexisting spherical refractive error could be useful for the investigation of meridional anisotropy related to astigmatic blur.

This study aims to characterize meridional anisotropy in contrast sensitivity (CS) and steady-state VEP (ssVEP) in myopic individuals with high WTR astigmatism. CS and ssVEPs were measured at spatial frequencies up to 12 cpd and compared with those from non-astigmatic individuals.

## 2 Methods

Thirty-four young adults, aged 18–35 years with a best-corrected distance visual acuity of logMAR 0 or better in each eye (measured using an EDTR chart), were recruited. This age range was selected to ensure reliable data, as the psychophysical CS measurements necessitate a comprehensive understanding of the testing procedures, which is more likely to be comprehended fully by adults. Individuals with clinically normal visual acuity were included to control for the confounding effects of amblyopia on CS and ssVEP responses (Shan et al., 2000; Davis et al., 2003; Baker et al., 2015). Previous findings indicate that meridional anisotropy can still persist in highly astigmatic individuals even with clinically normal visual acuity (Leung et al., 2021).

Participants were stratified into two groups based on their refractive status:

1. High astigmatic group (HAS): Spherical-equivalent error (SE) 0 to −6.00 D and cylindrical error (Cyl) ≥ 2.00 DC, with a negative cylindrical axis of 180° ± 20°.
2. Non-astigmatic group (NAS): SE ≥ −6.00 D and Cyl ≤ 0.50 DC.

Individuals with high myopia (SE < −6.00 D) were excluded due to the potential impact on VEP responses (Dani et al., 2020). This study focused on WTR astigmatism because it is the predominant astigmatic subtype in young Asian Chinese demographic (Chan et al., 2018; Leung et al., 2012; Wang et al., 2024). Those with anisometropia (i.e., difference in SE and Cyl between eyes) >1.00 D, strabismus, a history of ocular diseases, or eye surgery were excluded to minimize confounding variables. All participants underwent external and internal ocular health examinations and corneal topography to ensure they had regular corneal astigmatism and were free of ocular pathologies or corneal abnormalities, such as keratoconus.

Prior to the experimental procedures, each participant underwent a subjective refraction conducted by a registered optometrist to determine the full refractive-error correction required for CS and ssVEP assessments. The experiments were performed monocularly on the eye that met the refractive error criteria. If both eyes were eligible, the test eye was randomly chosen by the examiner. Refractive errors were fully corrected using spectacle trial lenses. The order of CS and ssVEP measurements was randomized for each participant.

The experimental procedures were approved by the Human Ethics Committee of The Hong Kong Polytechnic University (Approval No. HSEARS20210310003-01), and the research was conducted in accordance with the principles outlined in the Declaration of Helsinki. All experiments were performed only after obtaining the understanding and written informed consent of each participant.

### 2.1 Contrast sensitivity

CS was measured using Metropsis software (Cambridge Research Systems Ltd., UK) driven by a ViSaGe Stimulus Generator (14-bit RGB, Cambridge Research Systems Ltd., UK). Participants were seated 1.22 m from a gamma-corrected CRT monitor (Philips, Royal Philips, Netherlands) with a 1,264 × 949 display resolution and a 95 Hz refresh rate. The mean luminance was maintained at 49 cd/m^2^. Gabor stimuli with a sigma (*σ*) of 1° were presented at spatial frequencies of 0.6, 1.3, 3, 6, and 12 cpd for both vertically and horizontally oriented gratings.

The measurement employed a spatial four-alternative forced-choice procedure. In each trial, a Gabor stimulus was presented randomly in one of four quadrants on the screen, each with a spatial offset of 4.1° from the center. A 3-down-1-up modified staircase protocol was used to determine the contrast threshold. The initial contrast of the Gabor patches was set at 20% for spatial frequencies of 0.6, 1.3, and 3.0 cpd and 25% for 6.0 and 12.0 cpd. The initial step size in the staircase was set at 1.5 dB, with subsequent positive and negative step sizes of 0.3 and 0.5 dB, respectively. Participants were instructed to indicate the location of the Gabor patch by pressing the corresponding button on a response box. Audio feedback was provided to indicate the correctness of the response. The task was terminated after 12 reversals, with the threshold determined by the average of the last 8 reversals. Contrast thresholds were converted to logarithmic CS for analysis, calculated from the mean of two trials for each combination of orientation and spatial frequency. A parabola model (Robson, 1966) was fit to each participants’ contrast sensitivity function (CSF) to calculate the area under the log CSF (AULCSF) and spatial frequency cutoff using a second-degree polynomial (ax^2^ + bx + c) in R (v4.2.1, R Core Team).

### 2.2 Steady-state visual evoked potential

ssVEP was recorded in compliance with the guidelines established by the International Society for Clinical Electrophysiology of Vision (ISCEV) 2016 standard (Odom et al., 2016). The VERIS system (v6.0.6d19, Electro-Diagnostic Imaging, United States) was employed for the recordings. Pattern reversal sinusoidal gratings were presented with a mean luminance of 50 cd/m^2^ and an 11.1° square field size on a gamma-corrected LCD monitor (Samsung, Samsung Electronics Co., Ltd., Korea) with a 1,920 × 1,080 resolution and a 60 Hz refresh rate. The spatial frequencies of the gratings were 0.6, 1.3, 3, 6, and 12 cpd. The Michaelson contrast was set to 80%, and the stimulus pattern was reversed at a rate of 7.5 reversals per second (rps).

Gold cup electrodes, facilitated by conducting gel, were affixed according to the International 10–20 System (Klem et al., 1999). The active electrode was placed over the occipital cortex (Oz), the reference electrode on the forehead (Fz), and the ground electrode on the purlicue of the right hand. The impedances of the electrode-skin interface were maintained below 5 kΩ, with a maximum differential of less than 1 kΩ between measurements, as per ISCEV standards (Odom et al., 2016).

Measurements were conducted in a dimly lit room (illuminance <5 lux) to minimize distractions from the surroundings. Participants were positioned at a viewing distance of 150 cm from the monitor and instructed to maintain their gaze on a red central fixation cross (size: 0.8°) on the display throughout the recording session.

**Figure 1:**
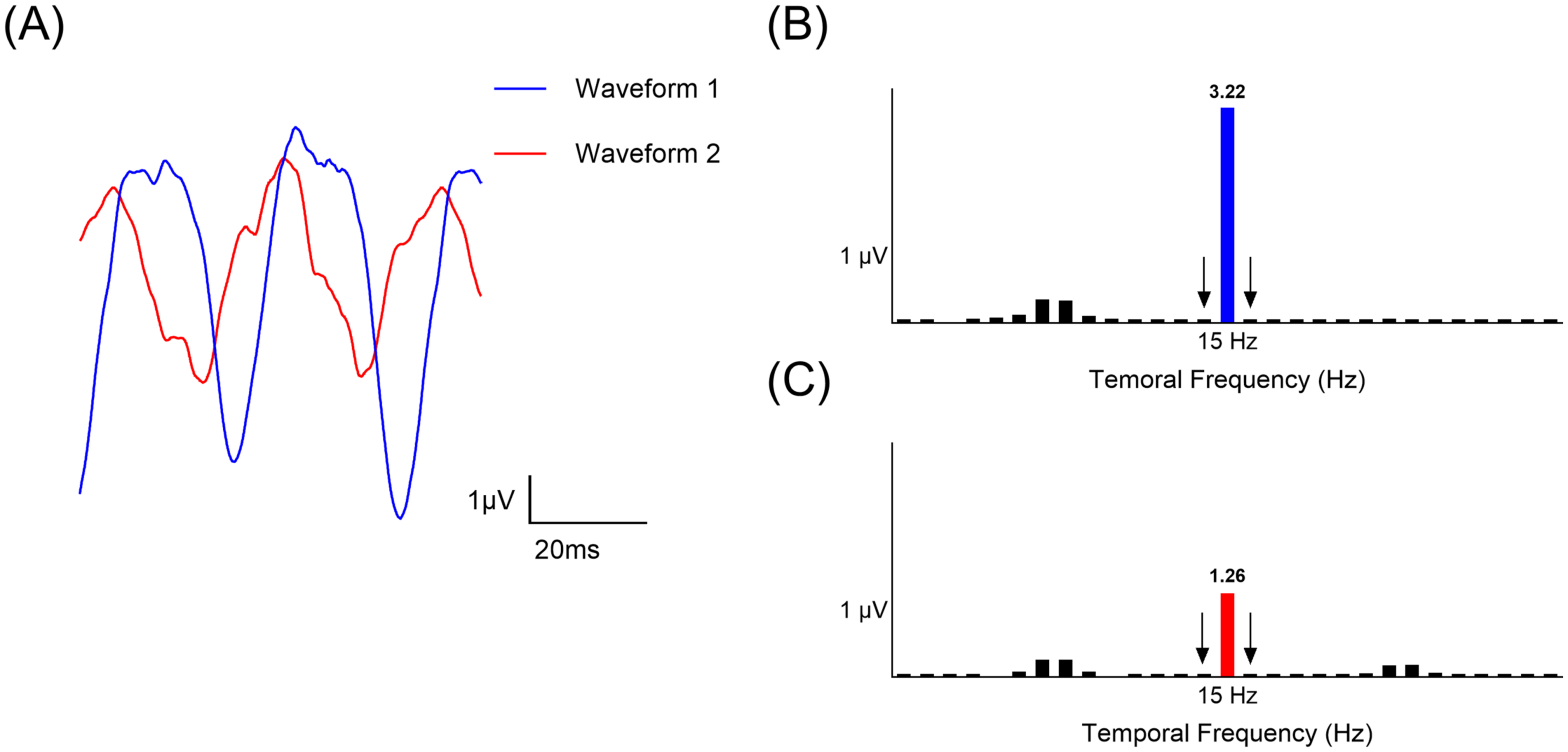
Analysis of steady-state Visual Evoked Potential (ssVEP) response. **(A)** Two unprocessed ssVEP waveforms. **(B)** Magnitude spectrum of waveform 1 (Blue) after Fourier transform. **(C)** Magnitude spectrum of waveform 2 (Red) after Fourier transform. The neighboring frequencies (14 Hz and 16 Hz, indicated by arrows) were used for signal-to-noise ratio calculation.

ssVEP responses were measured for each spatial frequency, along both the horizontal and vertical meridians, in a randomized sequence to control for order effects (Figure 1). The ssVEP signals were recorded by repeating four segments of measurements, each lasting 8.53 s, with breaks between each segment. Three trials were taken for each condition, with a total of 30 trials per subject. Subjects were instructed to fixate on a central fixation target and avoid blinking during the measurement periods. The recorded ssVEP signals were amplified and band-pass filtered between 3 Hz and 100 Hz. Additionally, a band-reject filter was applied to exclude 50 Hz electrical noise. The data were then analyzed using a discrete Fourier transform to obtain the amplitude at the stimulus frequency of 15 Hz. The noise level was determined by averaging the amplitudes at the neighboring frequencies of 14 and 16 Hz. A Signal-to-Noise Ratio (SNR) was calculated by dividing the signal amplitude by this average noise level. Trials with a SNR of 3 or higher were considered reliable and included in the analysis (Meigen and Bach, 1999). The percentage of trials rejected due to poor SNR was 0.19%. Analyses were performed using R (v4.2.1, R Core Team) with the eegkit package (v1.0-4) (Helwig, 2018). The geometric mean of the logarithmic VEP response amplitudes across the three trials were calculated for each spatial frequency and used for further analysis.

### 2.3 Statistical analysis

Statistical analysis was conducted using the JASP (v0.16.4.0). Unpaired t-tests were used to compare demographic information between the HAS and NAS groups. Mixed repeated measure ANOVAs were conducted to evaluate the effects of refractive group (between-subjects factor: HAS and NAS), spatial frequency (within-subjects factor: 0.6, 1.3, 3, 6, and 12 cpd), and orientation (within-subjects factor: horizontal and vertical) on CS and ssVEP responses. The Greenhouse–Geisser correction was applied when the assumption of sphericity was violated, as assessed by Mauchly’s test. To account for multiple comparisons across the five spatial frequencies tested, *p*-values were adjusted using the Holm-Bonferroni correction. Pearson’s correlation analyses were performed to evaluate the relationships between CS and ssVEP responses for each spatial frequency and orientation tested. The CS and ssVEP of individual participants are presented in Supplementary Figure S1.

## 3 Results

### 3.1 Demographic information

A total of 34 participants who fulfilled the inclusion criteria were initially recruited for the study. However, two participants from the NAS group dropped out due to the extended measurement time, resulting in a final sample size of 32 (16 HAS and 16 NAS). Table 1 summarizes the demographic information for the HAS and NAS groups for those who completed the entire study. As expected from the inclusion criteria, Cyl was significantly higher in the HAS group than the NAS group by 2.57 D (unpaired *t*-test, *t* = −8.91, *p* < 0.001). There were no significant differences in age (*t* = −0.34, *p* = 0.73) and spherical error (*t* = −1.85, *p* = 0.07) between the two groups. The spherical error, cylindrical error, axis and VA of the tested eye of the HAS and the NAS group are summarized in Supplementary Table S1.

**Table 1:** Demographic information of participants in the highly astigmatic (HAS) and non-astigmatic (NAS) groups (mean ± SEM [Range]).

	HAS (*n* = 16)	NAS (*n* = 16)	Overall (*n* = 32)
Age (years)	21.1 ± 0.5 [18.0, 26.0]	21.3 ± 0.2 [20.0, 24.0]	21.2 ± 0.3 [18.0, 26.0]
Spherical error (D)	−2.55 ± 0.41 [−4.25, 0.00]	−1.50 ± 0.39 [−5.00, 0.00]	−2.02 ± 0.29 [−5.00, 0.00]
Cylindrical error (D)	−2.80 ± 0.28 [−6.00, −2.00]	−0.234 ± 0.05 [−0.50, 0.00]	−1.52 ± 0.27 [−6.00, 0.00]

### 3.2 Contrast sensitivity

A mixed repeated measure ANOVA revealed a significant three-way interaction among refractive group, spatial frequency, and orientation (*F* = 2.95, Greenhouse–Geisser *p* = 0.045, η^2^*_p_* = 0.09). This indicates that the effect of spatial frequency and orientation on CS differed between the HAS and NAS groups.

**Figure 2:**
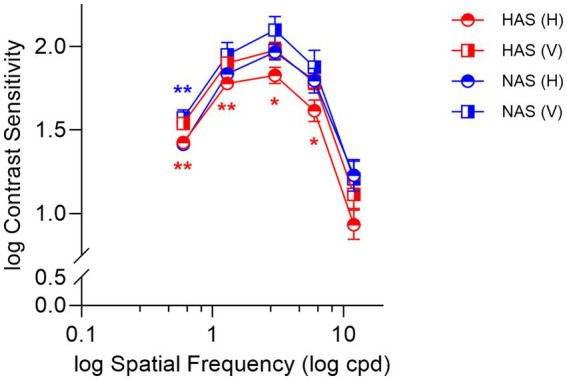
Mean ± SEM of log contrast sensitivity for the HAS and NAS groups at vertical and horizontal orientations across various spatial frequencies (cpd). Data from the HAS and NAS groups are represented in red and blue, respectively. Asterisks (*) represent significant differences in CS between horizontal and vertical orientations within the same group (corrected *p*-values: *p < 0.05, **p < 0.01).

Simple main effects analyses found that within the HAS group, CS was significantly lower for horizontally oriented gratings at spatial frequencies from 0.6 to 6 cpd (Figure 2, red symbols, *F* > 9.41, *p* < 0.009, corrected *p* < 0.045, η^2^*_p_* > 0.90). Conversely, such a meridional anisotropy in CS was not observed in the NAS group (Figure 2, blue symbols, *F* = 3.40, *p* = 0.085) except at 0.6 cpd (*F* = 15.09, *p* < 0.001, corrected *p* < 0.005, η^2^*_p_* = 0.94).

Comparing the refractive groups, the HAS group exhibited lower CS for horizontal gratings at 3 cpd (Simple main effects analyses: *F* = 4.22, *p* = 0.049, corrected *p* = 0.25, η^2^*_p_* = 0.81) and 12 cpd (*F* = 5.17, *p* = 0.030, corrected *p* = 0.15, η^2^*_p_* = 0.84) than the NAS group. However, these differences were not statistically significant after correcting for multiple comparisons.

### 3.3 AULCSF and spatial frequency cutoff

**Figure 3:**
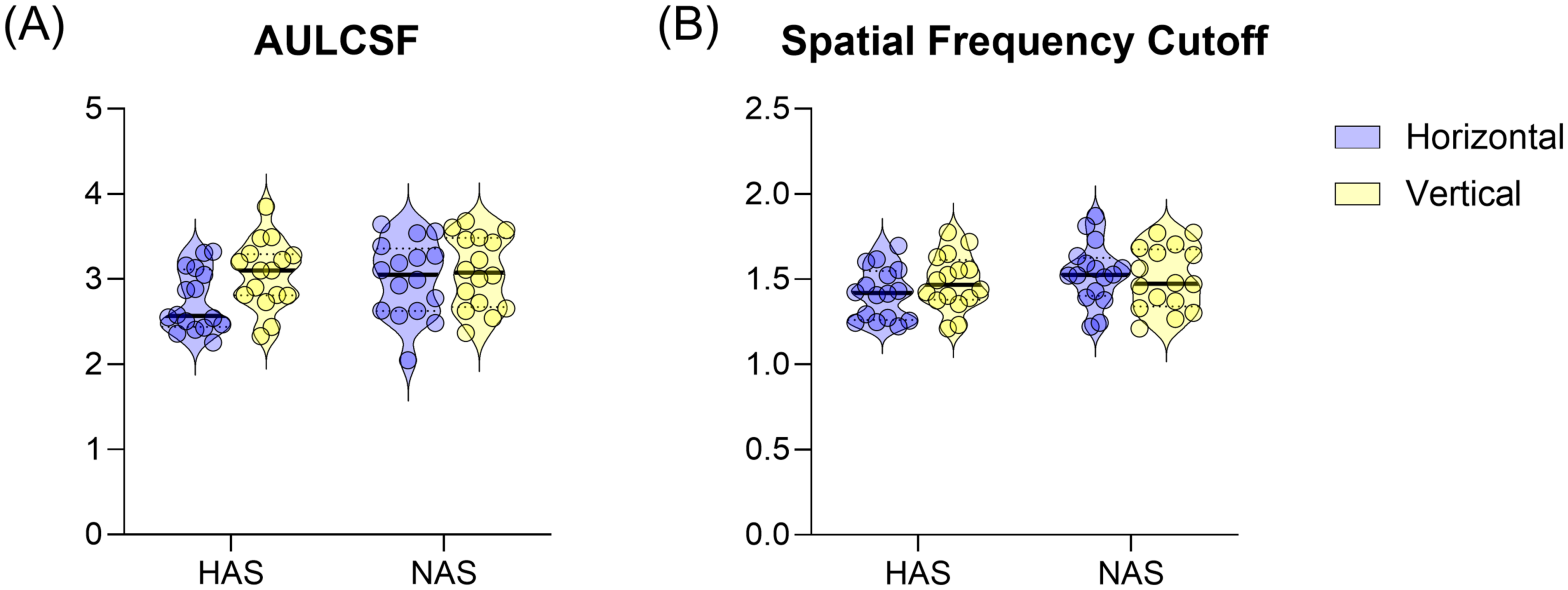
Mean ± SEM of **(A)** AULCSF and **(B)** spatial frequency cutoff for the HAS and NAS groups at vertical and horizontal orientations. Data from the Horizontal and Vertical are represented in Blue and Yellow, respectively.

A mixed repeated measure ANOVA on AULCSF revealed a significant main effect of orientation (*F* = 10.63, *p* = 0.003, η^2^*_p_* = 0.26), with lower AULCSF for horizontal than vertical gratings (*t* = −3.26, corrected *p* = 0.003). The main effect of refractive group was not significant (*F* = 1.26, *p* = 0.27, η^2^*_p_* = 0.04), nor was the interaction between refractive group and orientation (*F* = 3.42, *p* = 0.074, η^2^*_p_* = 0.102). Despite the non-significant interaction, planned comparisons using paired t-tests, with Bonferroni correction for multiple comparisons, were conducted to further explore the effect of orientation within each group. These revealed a significantly smaller AULCSF for horizontal than vertical gratings in the HAS group (Figure 3A, *T* = −3.28, corrected *p* = 0.01). This difference was not significant in the NAS group (*t* = −1.12, corrected *p* = 0.56).

For the spatial frequency cutoff, there was no significant interaction between refractive group and orientation (Figure 3B, *F* = 2.353, *p* = 0.135, η^2^*_p_* = 0.073). There were also no significant main effects of refractive group (*F* = 1.76, *p* = 0.195, η^2^*_p_* = 0.06) or orientation (*F* = 0.40, *p* = 0.532, η^2^*_p_* = 0.01).

### 3.4 Steady-state VEP responses

**Figure 4:**
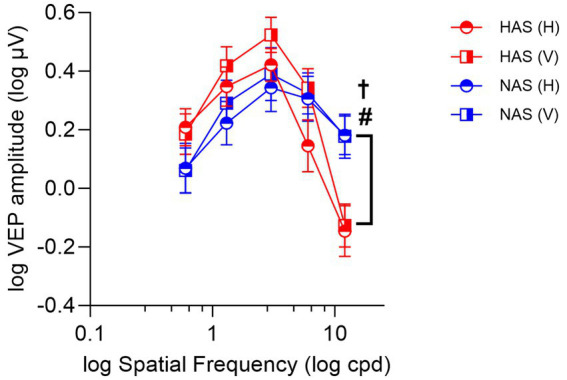
Mean ± SEM of log ssVEP amplitude for the HAS and NAS groups at vertical and horizontal orientations across various spatial frequencies (cpd). Data from the HAS and NAS groups are represented in red and blue, respectively. Octothorpes and obelus indicate significant differences in CS between refractive groups at horizontal (^#^*p* < 0.05) and vertical orientations (^†^*p* < 0.05).

A mixed repeated measure ANOVA examining the effects on ssVEP amplitudes revealed a significant interaction between refractive group and spatial frequency (*F* = 9.31, Greenhouse–Geisser *p* < 0.001, η^2^*_p_* = 0.237). Specifically, at the highest spatial frequency (12 cpd), ssVEP amplitudes were significantly lower in the HAS group compared to the NAS group, irrespective of grating orientations, as shown by simple main effect analyses (Figure 4, *F* = 6.19, *p* = 0.003; corrected *p* = 0.015, η^2^*_p_* = 0.92).

The analysis also revealed a significant main effect of orientation (*F* = 7.03, *p* = 0.013, η^2^*_p_* = 0.19). However, no significant interactions were found between orientation and either astigmatic group (*F* = 1.74, *p* = 0.20, η^2^*_p_* = 0.06) or spatial frequency (*F* = 2.19, Greenhouse–Geisser *p* = 0.10, η^2^*_p_* = 0.07). Generally, participants from both refractive groups exhibited higher ssVEP amplitudes for vertical orientations compared to horizontal.

### 3.5 Correlations between contrast sensitivity and steady-state VEP responses

Pearson’s correlation was used to examine the relationships between CS and ssVEP amplitude across various orientations and spatial frequencies within each refractive group. Significant correlations were found for the horizontal grating of NAS group at 3 and 12 cpd (*r* > 0.53, *p* < 0.03). However, after applying the Holm-Bonferroni’s correction for multiple comparisons, there correlations were no longer statistically significant (corrected *p* > 0.22).

## 4 Discussion

Consistent with existing literature (Mitchell and Wilkinson, 1974; Freeman and Thibos, 1975a; Gu et al., 2021), this study revealed meridional anisotropy in contrast sensitivity among individuals with high astigmatism, characterized by reduced sensitivity to horizontal gratings despite clinically normal visual acuity (logMAR 0 or better). This orientation-specific deficit aligns with the optical characteristic of myopic WTR astigmatism and persisted even with optical correction, suggesting reduced sensitivity of the visual system for horizontally oriented stimuli. Unexpectedly, ssVEP results showed no significant difference in meridional anisotropy between highly astigmatic and non-astigmatic groups.

This lack of ssVEP anisotropy contrasts with Fiorentini and Maffei (1973), who reported meridional anisotropy in transient VEPs in some children with high (Cyl: 3.00 to 4.00 D), but not mild (Cyl: 0.50 to 1.50 D), astigmatism. However, their limited reporting of visual acuity, ocular health, and quantitative VEP analysis hinders direct comparison. Our ssVEP findings align more closely with recent studies using transient and sweep VEPs (Yap et al., 2019; Yap et al., 2020; Gu et al., 2021), which also found meridional anisotropies (lower VEP amplitudes for horizontal than vertical gratings) in both astigmatic and non-astigmatic participants. However, these studies also had limitations: Yap et al. (2019) used a relatively low cutoff for defining astigmatism (Cyl ≥ 0.50 D) and did not investigate the impact of astigmatic magnitudes on VEP responses, while Gu et al. (2021) did not include a non-astigmatic control group, making it difficult to ascertain whether the observed meridional anisotropy is specifically associated with high astigmatism or a general visual processing characteristic. By comparing high myopic WTR astigmats to non-astigmatic controls, our study attempted to minimize the variability in astigmatic blur caused by different spherical refractive errors and astigmatism subtypes. Despite this targeted approach, our results indicate that meridional anisotropy in VEP responses is not exclusive to individuals with high astigmatism.

While we did not directly test the “horizontal effect,” our findings of reduced VEP responses to horizontal stimuli (compared to vertical stimuli) may be relevant to this body of research (Yap et al., 2024; Yap and Boon, 2020). This effect has been observed in visually normal preschool- and school-aged children, manifested as lower VEP amplitudes and longer latencies for horizontal gratings (Yap et al., 2019). This orientation bias may reflect an adaptation to the anisotropy of natural scenes, where perceptually discounting the prevalent horizontal content could enhance the salience of objects against a typical background (Hansen and Essock, 2004). However, because our study only tested horizontal and vertical gratings, we did not assess the oblique orientation and cannot rule out the “oblique effect” (Arakawa et al., 2000; Maffei and Campbell, 1970). Further research, including oblique orientations, is needed to determine whether our findings truly represent a horizontal effect, characterized by lower VEP responses to horizontal stimuli compared to all other meridians.

This study initially hypothesized that the meridional anisotropy in VEPs due to astigmatism would be more pronounced at higher spatial frequencies. However, our findings did not support this hypothesis. Indeed, meridional anisotropy in VEPs does not necessarily affect only higher spatial frequencies. Arakawa et al. (2000) investigated the oblique effect in non-astigmatic adults and observed a shift in the peak spatial frequency tuning for oblique stimuli toward lower spatial frequencies in ssVEPs, rather than a simple increase in the difference between cardinal and oblique orientations at higher spatial frequencies. This demonstrates that orientation bias in VEPs is complex and does not simply increase with spatial frequency, supporting the absence of a clear association between astigmatism and meridional anisotropy at higher spatial frequencies in our study.

This discrepancy between contrast sensitivity and VEP findings could be attributed to the distinct visual processing mechanisms underlying each test. Contrast sensitivity, a psychophysical measure, quantifies the minimum luminance contrast detectable by an individual (Owsley, 2003; Kim and Kim, 2010). In contrast, VEP provides an objective assessment of cortical activity, primarily reflecting the electrical potentials generated by the primary visual cortex in response to super-threshold visual stimuli (typically 50–80% contrast) (Kothari et al., 2016; Yap et al., 2019; Yap et al., 2020; Gu et al., 2021). It is possible that while visual impairments impact contrast sensitivity at threshold levels without much effect on VEPs at super-threshold levels in individuals with high astigmatism due to the involvement of different neural pathways or compensatory mechanisms at higher contrast levels. Additionally, contrast sensitivity involves more complex perceptual processes, such as attention and learning, which might not significantly correlate with the objective nature of VEPs.

Furthermore, the contrast sensitivity test employed in this study, similar to previous research, primarily assesses macular function, with target size usually less than 6° in diameter (Freeman and Thibos, 1975a; Gu et al., 2021; Mitchell and Wilkinson, 1974). Conversely, VEP potentially integrates both macular and paramacular responses, with target sizes ranging from 10° to 12° (Yap et al., 2019, Yap et al., 2020, Gu et al., 2021). Notably, beyond the macular region, the visual system exhibits an orientation bias favoring radial orientations (Zheleznyak et al., 2016; Leung et al., 2021). For example, the horizontal visual field is more sensitive to horizontally oriented gratings, while the vertical field is more attuned to vertically oriented gratings. This inherent radial bias in the paramacular region, captured by the larger VEP stimulus, might dilute the impact of the macular-dominated meridional anisotropy observed in contrast sensitivity tests.

Our study observed a somewhat unexpected trend toward higher VEP responses at lower spatial frequencies in high astigmats compared to non-astigmats (Figure 4), whereas psychophysical contrast sensitivity at these frequencies was similar or even slightly reduced in the high astigmatism group compared to the non-astigmatic group (Figure 2). The difference in methodologies between the VEP (pattern-reversal grating flickering at 7.5 rps) and psychophysical (steady grating) measurements may contribute to this discrepancy. Specifically, the magnocellular pathway is known to be more sensitive to temporal modulation (such as flicker) and lower spatial frequencies (Lee et al., 1990; Smith et al., 2001). Therefore, the VEP responses, particularly at lower spatial frequencies, may also be influenced by magnocellular activity with the flickering stimulus used in this study. However, further research is needed to determine whether the observed trend toward increased VEP responses in high astigmats represents a genuine difference in magnocellular function. It is important to acknowledge that the limited sample size in our study may mean the observed trend could be due to chance. Further research with a larger sample size is warranted to confirm our observations.

While this study contributes to the growing body of evidence suggesting that distinctive meridional anisotropy is absent in VEP measurements of high astigmatism, several limitations need acknowledgment. First, we solely focused on participants with myopic WTR astigmatism due to its predictable meridional anisotropy (Mitchell et al., 1973; Harvey et al., 2007; Harvey, 2009) and prevalence in the studied population (Chan et al., 2018; Leung et al., 2012; Wang et al., 2024). Excluding hyperopes was necessary to minimize variability in astigmatic blur (Harvey, 2009; Mitchell et al., 1973). However, these selective recruiting criteria may limit the generalizability of our results to these astigmatic subtypes.

Second, our study focused on participants with a clinically normal visual acuity (logMAR 0 or better). Therefore, our results may not generalize to individuals with impaired acuity due to high astigmatism (Yap et al., 2020; Gu et al., 2021; Yap et al., 2019). Lastly, our study involved adult participants, and their history of spectacle correction during childhood is undocumented. The meridional anisotropy observed in participants with high astigmatism might result from uncorrected or inadequately corrected astigmatism during the critical developmental period for orientation selectivity (Cobb and Macdonald, 1978; Gwiazda et al., 1986). After the age of three, optical correction tends to be less effective at mitigating meridional anisotropy (Harvey et al., 2008). Further research including young children could provide valuable insights into the development of meridional anisotropy, though the validity and reliability of psychophysical testing in such young cohorts would require careful consideration.

In conclusion, this study reveals that while meridional anisotropy in contrast sensitivity is present in young adults with high astigmatism, it is not consistently reflected in ssVEP measurements. Our findings, consistent with recent VEP studies (Yap et al., 2019; Yap et al., 2020; Yap et al., 2021; Gu et al., 2021), highlight the complex relationship between subjective visual performance and objective VEP responses. The discrepancy between subjective and objective measures underscores the complex, multi-level processing within the visual system and requires further investigation. Further research, potentially utilizing neuroimaging techniques or longitudinal designs, is essential to unravel the mechanisms underlying the discrepancies in functional and electrophysiological visual performance across different meridians in astigmatic eyes.

## Data Availability

The raw data supporting the conclusions of this article will be made available by the authors, without undue reservation.
