# Supplementary material for: Meridional anisotropy in contrast sensitivity and visual evoked potential in adults with high myopic astigmatism
Source: Front Neurosci. 2025 Jan 9;18:1457297. doi: 10.3389/fnins.2024.1457297 (PMC11754223; doi:10.3389/fnins.2024.1457297)
Supplement: Supplementary file 1 [file Data_Sheet_1.DOCX]

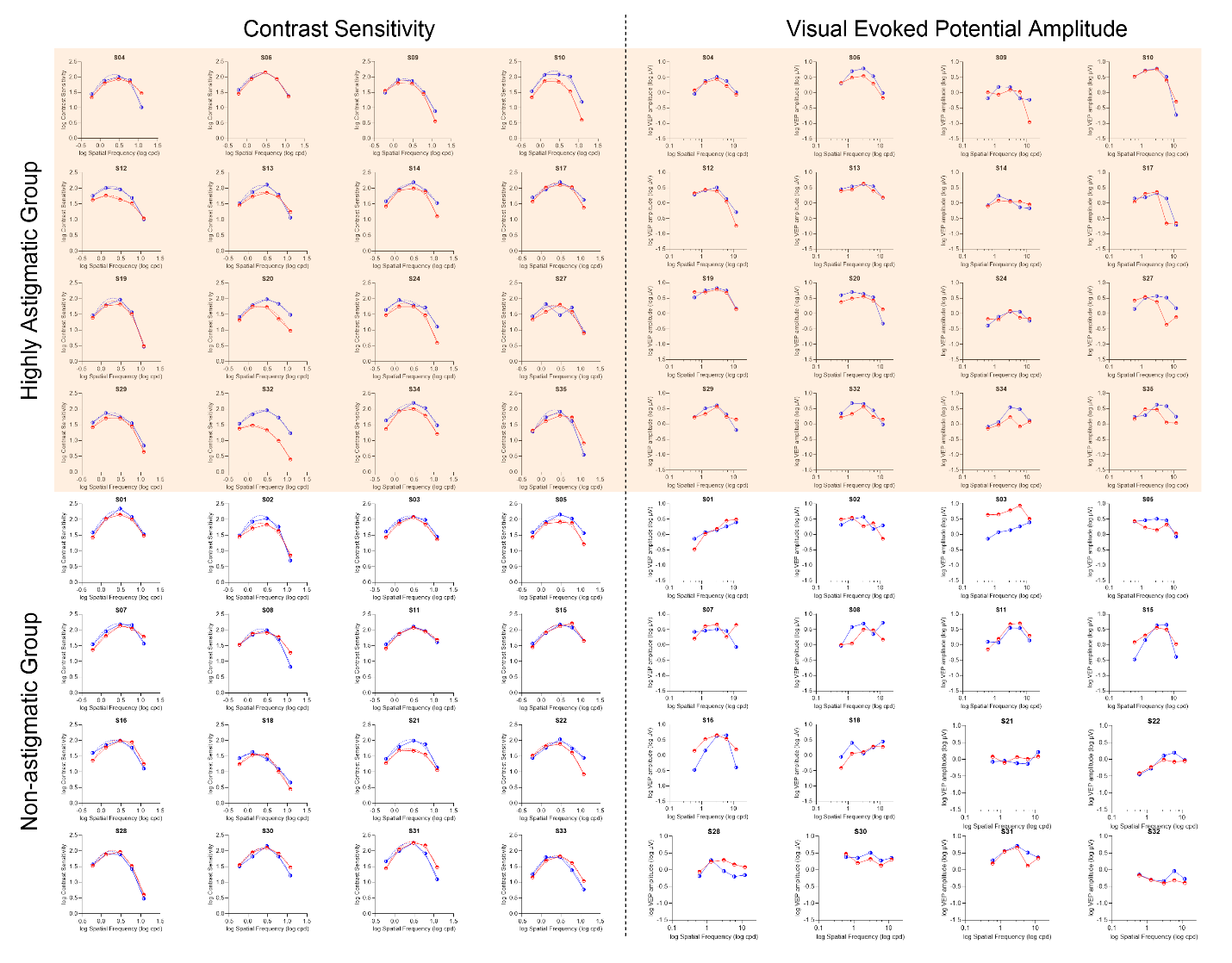


Supplementary Figure 1. Contrast Sensitivity and visual evoked potential amplitude for each participant. Red and blue data points represent horizontal and vertical gratings, respectively. Dashed lines represent second-order polynomial fits to the contrast sensitivity data. These fits were not applied to the VEP data due to poor fit quality.
