# Supplementary material for: Meridional anisotropy in contrast sensitivity and visual evoked potential in adults with high myopic astigmatism
Source: Front Neurosci. 2025 Jan 9;18:1457297. doi: 10.3389/fnins.2024.1457297 (PMC11754223; doi:10.3389/fnins.2024.1457297)
Supplement: Supplementary file 2 [file Table_1.DOCX]

| ID | Group | Sph (D) | Cyl (D) | Axis | VA (logMAR) |
| --- | --- | --- | --- | --- | --- |
| 04 | HAS | -0.25 | -3.00 | 175 | 0.00 |
| 06 | HAS | -3.75 | -2.00 | 175 | -0.06 |
| 09 | HAS | -0.25 | -6.00 | 177 | 0.00 |
| 10 | HAS | -1.50 | -3.50 | 5 | -0.10 |
| 12 | HAS | -3.25 | -2.00 | 165 | -0.08 |
| 13 | HAS | -0.50 | -2.50 | 175 | -0.10 |
| 14 | HAS | -3.00 | -2.00 | 160 | -0.06 |
| 17 | HAS | -1.00 | -4.25 | 12 | -0.04 |
| 19 | HAS | -4.50 | -2.25 | 175 | 0.00 |
| 20 | HAS | -3.50 | -2.00 | 170 | -0.08 |
| 24 | HAS | -4.25 | -3.25 | 175 | 0.00 |
| 27 | HAS | -3.50 | -2.00 | 178 | 0.00 |
| 29 | HAS | -3.50 | -2.00 | 180 | 0.00 |
| 32 | HAS | 0.00 | -3.75 | 175 | 0.00 |
| 34 | HAS | -4.00 | -2.25 | 168 | 0.00 |
| 35 | HAS | -4.25 | -2.00 | 180 | 0.00 |
| 01 | NAS | -3.00 | -0.50 | 175 | -0.08 |
| 02 | NAS | -0.75 | -0.50 | 15 | -0.08 |
| 03 | NAS | -0.75 | 0.00 | / | -0.10 |
| 05 | NAS | -0.25 | -0.25 | 20 | -0.08 |
| 07 | NAS | -1.00 | 0.00 | / | -0.12 |
| 08 | NAS | -5.00 | -0.50 | 180 | -0.10 |
| 11 | NAS | -0.50 | -0.25 | 175 | -0.08 |
| 15 | NAS | 0 | 0 | / | -0.16 |
| 16 | NAS | -4.75 | 0 | / | -0.08 |
| 18 | NAS | -1.25 | -0.25 | 10 | -0.06 |
| 21 | NAS | -1.00 | -0.25 | 180 | 0.00 |
| 22 | NAS | -0.25 | -0.50 | 140 | 0.00 |
| 28 | NAS | -1.25 | -0.50 | 165 | 0.00 |
| 30 | NAS | -2.00 | 0.00 | / | 0.00 |
| 31 | NAS | 0.00 | -0.25 | 15 | -0.10 |
| 33 | NAS | -2.25 | 0.00 | / | 0.00 |

**Supplementary Table 1.** Spherical refraction (D), Cylindrical refraction (D), Axis and Visual Acuity (logMAR) of the HAS and the NAS group.
